# Portrayal of Deaf characters in Korean movies

**DOI:** 10.1093/jdsade/enaf003

**Published:** 2025-01-22

**Authors:** Andrew Chang, Debbie Golos

**Affiliations:** Department of Special Education, Vanderbilt University, Nashville, TN, United States; Department of Educational Psychology, University of Minnesota, Minneapolis, MN, United States

## Abstract

Characters in movies have the potential to influence perceptions of how people see themselves. Deaf adolescents who have little opportunity to interact with Deaf peers or family members may be particularly drawn to Deaf people they see in the media. How the media portrays Deaf people may impact Deaf adolescents’ self-perceptions of and language preferences. Yet little is known about the ways Deaf people are portrayed in movies, particularly films developed outside of the United States and not in English. In this study, we utilized content analysis to explore the portrayal of Deaf characters in Korean movies and Deaf involvement in the production of films. Descriptive statistics were used to determine the extent to which characters were portrayed by medical or cultural perspectives of Deaf people. Findings indicate that 68.5% of scenes in the sampled Korean films include medical rather than cultural messages about Deaf people. Additionally, none of the movies had Deaf people directly involved in the production of the films. Implications for future directions regarding identity and portrayal of Deaf people in media are discussed.

As online streaming services such as Netflix and Disney Plus grow globally, many adolescents have more opportunities to experience various movies. Since adolescence is a period when they are questioning their identities (Baumeister & Tice, 1986; Erikson, 1968), characters in the movies they view have the potential to impact their identity development. In particular, Deaf^1^ adolescents, who have little opportunity to interact with Deaf adults or Deaf peers in their daily lives, may see Deaf characters in the movies as Deaf role models. In other words, how Deaf characters are portrayed in movies could potentially impact how Deaf adolescents view themselves as Deaf people and perhaps even their language preferences such as spoken or sign language (De Clerck & Golos, 2020; García-Fernández, 2014). This is true at a global level and includes Deaf Korean adolescents. As little is known about the portrayal of Deaf characters in media in Korea, it is beneficial for educators, researchers, and caregivers to understand the types of messages about Deaf people in Korean movies to make informed choices about the resources they recommend or view with adolescents.

## Importance of role models

Role models are strong drivers of a human’s growth (Bandura & Walters, 1977; Riley, 2022). They emphasize the importance observation plays, particularly when children or adolescents interact with people they see as role models. While growing up, humans build their self-efficacy as they see people of similar backgrounds with them successfully handle their work (Zeldin et al., 2008). Additionally, they begin to form identities through relationships with their role models (Kim et al., 2018; Stone, 1990), and these interactions may also support language acquisition. That is to say, role models may be essential for developing children’s social and linguistic awareness and sense of self (Bandura & Walters, 1977; Kim et al., 2018).

### Context of Deaf people in South Korea

As children grow up, role models can include parents, families, teachers, peers, friends, and media (Bandura & Walters, 1977; Bornstein & Bradley, 2014). However, as 90% of Deaf children come from hearing parents with little access to sign language and Deaf people (e.g., Mitchell & Karchmer, 2004), many need access to additional linguistic and cultural role models, including Deaf role models.

This is also true for Deaf children in South Korea as about 90% of Deaf people in Korea have hearing parents (Chang et al., 2013). This means that most Deaf children could grow up in families where they have little access to Deaf people and where Korean Sign Language (KSL) is not the primary mode of communication in the home. In addition, 76.1% of Deaf students in South Korea go to mainstream schools where Deaf role models and sign language are rarely found (Korean Ministry of Education, 2018). This suggests that the conventional educational system might not sufficiently cater to the cultural and linguistic needs of Deaf students, who could greatly benefit from having access to Deaf role models. The lack of exposure to cultural and linguistic role models from home and school can result in language delays and have significant effects on their communication abilities (De Clerck & Golos, 2020; García-Fernández, 2014), social interactions (Cawthon et al., 2016), and sense of self-identity (Sutton-Spence, 2010).

Furthermore, according to the Korea Press Promotion Foundation (2020), only 1.8% of Deaf students in Korea graduate from a Deaf program. This draws attention to the insufficient availability of education tailored to the specific needs of Deaf students that accommodates their distinct learning styles and fosters their overall well-being. This absence could contribute to the challenges these students face in finding relatable role models.

Namely, most Deaf students in South Korea do not have access to teachers and/or peers, as their Deaf role models. This means that many Deaf students in South Korea may not have the opportunity to learn about a larger Deaf community. As many South Korean Deaf adolescents may not be learning about the languages and cultural heritages of Deaf people from their homes and schools, this has the potential to lead to struggles with their social identities during adolescence. Therefore, having a better understanding of ways Deaf children can access role models could be important in creating a more inclusive and empowering environment for Deaf people in Korea.

### Importance of media

As Deaf adolescents in Korea may lack access to the Korean Deaf communities or Deaf role models from families, teachers, or peers, they may turn to the media for role models (Valkenburg & Piotrowski, 2017). Media, the main means of mass communication, comes in many forms including accessing print, video, and movies online or in person (Korea Press Promotion Foundation, 2020). Among them, Korean adolescents are highly dependent on video media. According to the Korea Press Promotion Foundation’s “2019 Survey on the Teen’s Use of Media” (2020), 87.4% of teenagers use video media more than once a week (e.g., YouTube, Netflix). Of them, 34.2% said they watch movies once a week. This suggests that there is potential for Deaf people in movies to be role models for Deaf adolescents in South Korea and possibly influence their identities (Riley, 2022).

Media has the potential to positively or negatively impact Deaf adolescents’ perceptions of self and others. While having a better understanding of how Deaf characters in media are portrayed, particularly at a global level, is important, most research about the representation of Deaf characters has focused on media in English (e.g., Golos et al., 2021; Golos, 2010; Foss, 2014; Wolfe-Webb, 2021). Little is known as to how Korean movies portray Deaf characters. Therefore, analyzing the portrayal of Deaf characters in Korean movies may be beneficial to have a better understanding of what Korean Deaf adolescents are viewing and also inform media development for Korean Deaf adolescents.

## Theoretical perspectives

### Social and cultural capital and media representation

The concepts of social and cultural capital may offer valuable insights in this area (Byatt et al., 2023; Kurz et al., 2021). Social capital refers to the resources available through social networks, while cultural capital includes knowledge, skills, education, and advantages that a person has, which give them a higher status in society (Ostrom, 2009). For Deaf individuals, social capital can be enhanced through connections within their immediate family and interactions including with Deaf people in their community, and cultural capital can be enriched through access to learning about Deaf culture and language (including sign language) at home, school, and environment, which may include through media.

Having social and cultural capital supports identity formation of Deaf adolescents (Byatt et al., 2023; Greene-Woods et al., 2020). Within the Deaf community, having social capital helps adolescents develop a strong Deaf identity by connecting them with role models and peers who share similar experiences. This connection reinforces their cultural identity and self-esteem (Byatt et al., 2023; Cawthon et al., 2016). Meanwhile, cultural capital helps reinforce the cultural identity of Deaf individuals by connecting them with the norms, language, and history of the Deaf community. This connection helps them affirm their identity, even in environments dominated by hearing norms (Greene-Woods et al., 2020).

In addition, social and cultural capital plays a role in the well-being and resilience of Deaf adolescents (Byatt et al., 2023; Greene-Woods et al., 2020). The support systems formed through bonding social capital contribute significantly to their emotional well-being by providing a safe space where they can express themselves and seek advice and understanding. Bridging social capital, by exposing Deaf adolescents to diverse experiences and perspectives, enhances their resilience in facing challenges related to being a Deaf person in a predominantly hearing world (Byatt et al., 2023). On the other hand, cultural capital itself may not directly build resilience. However, it provides a foundation of cultural knowledge and identity that supports other forms of capital, like navigational and resistance capital (Greene-Woods et al., 2020). This includes Deaf people developing strategies to utilize linguistic repertoires for navigating communication and interactions with individuals in different social contexts (De Meulder et al., 2019; Holcomb et al., 2023).

Despite these benefits, there are challenges in developing social and cultural capital for Deaf adolescents. Barriers such as lack of access to language, discrimination, bullying, and a lack of understanding from the hearing community can hinder their ability to build and maintain these valuable networks (Byatt et al., 2023; Cawthon et al., 2016). Addressing these challenges requires concerted efforts to promote inclusivity and understanding within educational and social institutions. With barriers to social and cultural capital, Deaf adolescents may rely more on media for self-understanding, and this may lead to potentially overinternalizing the messages they see in movies.

Some researchers have utilized two perspectives about deafness and Deaf people to guide their research: the medical view and the cultural view (e.g., Golos & Moses, 2011). The medical perspective focuses on improving individuals’ hearing levels rather than their sociocultural needs (Phillips, 1990). This views deafness as a deficiency. Also, it views the Deaf person as having a disability that needs to be fixed (Lane, 1992). This perspective emphasizes improving the hearing level and argues for the need for a hearing aid or a cochlear implant. Proponents of this model generally recommend Deaf children to use spoken language and may not recommend sign language. They may not highly value Deaf cultures, Deaf communities, and natural sign languages (e.g., ASL, KSL) (Parasnis, 1996).

In contrast, others view Deaf people from a cultural perspective (Jones, 2002; Kurz et al., 2021; Ladd, 2003) Deaf culture refers to the set of attitudes and beliefs that uphold the core values of Deaf communities, such as full access to communication, information sharing, and self-determination (Ladd, 2003). Proponents of this perspective recognize the role and importance of social and cultural capital so that Deaf people can live rich and fulfilled lives particularly when they have access to language and Deaf people whether or not they choose to use hearing aids CI or other technologies. They also recognize the value of Deaf schools that provide access to language and a nurturing environment to share the culture and traditions of Deaf people with Deaf students (Ladd, 2024; The National Association of the Deaf, 2021; Lane et al., 1996).

Taking into consideration the medical and cultural perspectives of Deaf people along with social and cultural capital provides a framework for analyzing the portrayal of Deaf characters in Korean movies. When these perspectives are viewed through the lens of social and cultural capital theories, these portrayals might not just affect the individual identity of viewers; they also could influence the collective resources and status of Deaf people in Korean society. For example, a film that predominantly includes only medical messages may undermine the social capital of Deaf individuals by reinforcing stereotypes and limiting opportunities for social inclusion. Conversely, films that portray cultural messages of Deaf people may enhance cultural capital by promoting awareness and appreciation of Deaf culture and sign languages, thus fostering greater social inclusion and identity affirmation among Deaf adolescents.

## Literature review

Researchers have examined the potential influence of Deaf role models and media representations on the identity, self-esteem, and social capital of Deaf individuals. The messages of how Deaf people have been portrayed in print and video media have also been explored, although, primarily within the United States, there are emerging studies beginning to explore media on a global level.

### Role models and Deaf people

By observing positive role models, Deaf people may build their self-esteem, appreciate their value to society, and utilize their multilingual/multimodal abilities. Observing positive role models may also have a positive impact on their potential academically and socially (Cawthon et al., 2016; Holcomb, 1997; Sutton-Spence, 2010). Cawthon et al. (2016) conducted a research synthesis of 18 studies to explore the role of Deaf role models in building social capital. The studies focused on various forms of support, including role model programs and personal guidance, highlighting their contributions to developmental processes and social connections for Deaf individuals​. The authors concluded that positive role models help Deaf individuals build social capital by offering crucial support and guidance, fostering both academic resilience and social growth. This support empowers Deaf children to navigate societal barriers with greater confidence.

In addition, they may be positively influenced linguistically by observing their role models engage in successfully fluent and accessible communication such as in sign languages (De Clerck & Golos, 2020; García-Fernández, 2014). According to Scott (2022), early access to a fully accessible language like sign language (here American Sign Language; ASL), can provide Deaf children a foundation for future reading success. Thus, in addition to positively impacting their self-esteem, Deaf people with access to language role models may positively impact their language skills as well.

The identities of Deaf people may play an essential role in even their language choices (De Clerck & Golos, 2020; Gulati, 2019). For example, Park et al. (2012) conducted a phenomenological study involving four Deaf adolescents in Korea to examine their Deaf identity formation and found that all four participants were initially influenced by medical perspectives, which led to negative self-perceptions and resistance to learning sign language. However, as some participants were later exposed to sociocultural perspectives, such as Deaf culture and community, their identity evolved positively, and they began to embrace sign language. Similarly, if Deaf child is influenced by cultural messages, they may be more likely to have a positive identity and feel pride about themselves as a Deaf person, Deaf culture, and other Deaf people and may prefer sign language as their primary mode of communication (Parasnis, 1996). Therefore, the languages a Deaf person prefers to use may be connected to how they identify themselves.

Korean Deaf adolescents may not be likely to not identify with Deaf people or Deaf communities because, as mentioned, Deaf adolescents in Korea are not frequently exposed to Deaf people or KSL at home or school (Choi, 2020). This, in turn, may not provide them an opportunity to experience a rich Korean language environment, and this may result in them experiencing language delays or deprivation because they were not provided with a fully accessible language from birth (Glickman & Hall, 2018). However, even if Deaf children in Korea were not exposed to Deaf people in childhood, they can develop a positive Deaf identity by encountering positive Deaf role models throughout their childhood (Cawthon et al., 2016). If Deaf adolescents observe and interact with diverse Deaf people in the community, including through media in school and society, it may increase their positive sense of self as a Deaf person.

### Portrayal of Deaf people in media and print

As mentioned, limited studies have examined Deaf people in the media. Prior studies that have analyzed the portrayal of Deaf people in media are limited to analysis of media in English. As limited studies examine film and digital media, portrayal of Deaf characters in literature also provides insight into how Deaf people are portrayed more broadly. Findings from these studies in both areas highlight the importance of providing children from multicultural backgrounds with role models who share similar linguistic and cultural backgrounds.

For example, in an analysis of written text in children’s literature, Golos and Moses (2011) analyzed 20 picture books that included deaf characters and for children ages 4–8 years for messages linked to medical and cultural categories. The findings revealed that the majority of messages in these books did not portray Deaf characters from a cultural perspective. Instead, they focused on features of deafness as a medical condition to be fixed, perpetuating perceptions of deafness as a disability.

In a follow-up study, Golos et al. (2021) examined the illustrations of these same books, comparing the messages they previously found in the written text. According to the findings, the illustrations similar to the text more frequently portrayed deaf characters from a medical perspective, and there were sometimes mixed or conflicting messages in the illustrations and text on the same page. From both these studies, they suggested that the messages in the books may have been impacted by the majority of authors being hearing and with little input from Deaf people in the writing or illustration of the book. They recommended that more books with Deaf authors, illustrators, and consultants are needed.

In regards to books targeted to adolescents, Gangwish (2017) examined 20 young adult novels that featured deaf or hard-of-hearing teenagers released between 2000 and 2017. They used a qualitative content analysis method to gain a better understanding of how Deaf characters and Deaf culture are represented in modern young adult literature. Throughout the 20 novels examined, they found that medical perspectives were the most prevalent, emphasizing not only the pathological aspects of deafness but also the social isolation that Deaf people experience. These findings indicate that the sampled modern young adult novels present Deaf individuals as characters who need to be fixed rather than valuing them for who they are.

There are a few studies that have analyzed deaf characters in video media. For example, Golos (2010) examined the portrayal in educational television programming for children in the United States and found a pattern of portraying Deaf people from a medical perspective. Foss (2014) explored television more broadly, examining the changing representations of deafness and Deaf culture on 40 television programs between 1987 and 2013. While some improvements have been made, such as storylines promoting tolerance and acceptance of Deaf culture and the use of ASL, they concluded that the pathological model still is more prevalent, depicting Deaf characters as vulnerable or dehumanized and advocating cochlear implants as a “cure” for deafness (Foss, 2014). The portrayal of ASL as a language often falls short, lacking in capturing its grammatical complexity. These depictions create the perception that deafness is socially constructed, influenced by context and experiences, reflecting real-life tensions between the Deaf community and medical experts. To challenge stigmas and promote mainstream acceptance of Deaf culture, Foss (2014) emphasizes the importance of increased diversity in roles for Deaf actors on television.

More recently, Wolfe-Webb (2021) studied how sign language and Deaf culture are portrayed in mainstream films in the United States by using a mixture of textual and discourse analysis. They analyzed three films: *Children of a Lesser God, Hush*, and *A Quiet Place*. In regards to the movie, *Children of a Lesser God*, while it cast a Deaf actress and won an Academy Award and Golden Globe, Wolfe-Webb (2021) noted that this movie perpetuates stereotypes by portraying Deaf people as disabled, broken, or in need of fixing. Also, Wolfe-Webb (2021) concluded that *Hush* received recognition and positive reviews from hearing audiences on Netflix. However, the findings indicate that the film falls short in appropriately representing Deaf culture, as it lacks both proper casting of Deaf actors and meaningful consultation with Deaf individuals, resulting in an inaccurate portrayal of Deaf technology. In contrast, Wolfe-Webb (2021) highlighted *A Quiet Place* as an example of successful and inclusive representation, commending its critical acclaim and the production’s efforts to involve Deaf people in both consultation and acting roles. This underscores Wolfe-Web’s argument about the importance of including Deaf individuals throughout the filmmaking process to achieve authentic representation.

While these studies provide some insight into the portrayal of Deaf people in printed and video media, they focus on movies created and produced in English. Diffrient (2017) conducted a qualitative textual analysis of three contemporary South Korean films: *Silenced* (2011), *Always* (2011), and *Blind* (2011)—focusing on how these films portray disability, which included examining deafness. Diffrient (2017) highlighted a prevalent trend in Korean films to frame disability through a medical model, often portraying characters with disabilities as helpless or childlike. He mentioned that the movie *Silenced* stands out by critiquing societal silence and discrimination, shedding light on abuse within a school for Deaf students, however, it still reinforces infantilizing portrayals by focusing on hearing adults as saviors while marginalizing Deaf characters as passive figures in their own story. Diffrient (2017) argued that while these films raise important awareness, they often fall short of granting disabled characters full agency, inadvertently reinforcing stereotypes that can hinder societal understanding and inclusivity. This lends itself to also considering overall plot messages of a storyline in addition to messages within the movie.

Although Diffrient’s (2017) study highlighted trends in Korean disability portrayals, it largely centers on general disability representation, with only one film featuring Deaf characters. This limited scope makes it difficult to identify broader trends specific to Deaf portrayals in Korean movies and the overall storyline. This paper addresses this gap by examining Deaf portrayals in Korean films, providing insights into the types of messages conveyed to Deaf viewers in Korea and future considerations.

### Purpose

The purpose of this study was to examine the portrayal of Deaf characters in Korean Movies. The research questions guiding this study are: (a) In what ways do Korean movies portray Deaf characters from a medical perspective? (b) In what ways do Korean movies portray Deaf characters from a cultural perspective? (c) What are the overall messages about Deaf people portrayed by the overall storyline? and (d) To what extent are Deaf people involved in the development and production of Korean movies that include Deaf characters?

## Methods

In this study, a content analysis was utilized for the portrayal of Deaf characters in Korean movies released between 2010 and 2024. Guided by Krippendorff’s methodologies for content analysis (Krippendorff, 2018), the research team applied a comprehensive set of selection criteria to identify trends and patterns in how Deaf characters are represented. This involved coding for cultural and medical messages to identify trends both within and across movies as well as identifying the extent of involvement of Deaf people in the filmmaking process.

### Sample selection

While past studies have examined all Deaf characters (Gangwish, 2017; Golos & Moses, 2011), in this study we considered movies where the Deaf character was a main character to be able to also examine the overall story plot. The inclusion criteria included: The movie must clearly state that the character is Deaf. Movies that do not clearly state that a character is Deaf were excluded.

- The Deaf characters need to be the main characters with their own plotlines to ensure a more in-depth analysis of their representation.
- The movie must have been released after 2010 to reflect more current cultural perspectives.
- The length of the movie must be at least one hour as a typical full-length feature film.
- The movie must not be rated NC-17 to ensure that it is appropriate for teenagers as we were exploring movies for adolescents.

To identify movies that met selection criteria, the lead author conducted a comprehensive search using Google, Naver, and Daum to identify Korean movies released from January 1, 2010, to February 28, 2024. The search focused on movies with the highest number of Korean audiences according to the Korean Box Office Information System. Keywords “Korean movies Deaf character,” “Movies with Deaf characters,” and “Korean movies with Deaf representation” were used. After compiling a list of movies, the author reviewed plot summaries, cast lists, and reviews to identify films featuring Deaf characters. Each shortlisted movie was watched to confirm that Deaf characters had significant roles with their own plotlines. In addition, the movies had to be clearly stated as having Deaf characters, released post-2010, at least 1 hour long, and not rated NC-17. This process narrowed the six Korean movies: *“Along with the Gods: Two Worlds,” "G-Love," “Like for Likes,” "Midnight," "Silenced," and “You’re So Precious to Me.*”

### Plot of each movie

In *Along with the Gods: Two Worlds*, firefighter Ja-hong undergoes seven trials in the afterlife, reflecting on unresolved family issues with his Deaf mother. As a teenager, Ja-hong attempted a murder-suicide to end his mother’s suffering, which stemmed from poverty and illness, but he failed and fled home in guilt. Fifteen years later, he receives his mother’s forgiveness, mending their relationship. Throughout the entire film, the mother is depicted as frail, impoverished, and reliant on others.

In *G-Love,* a hearing baseball coach takes on a team of Deaf players and grows closer to them as they break stereotypes in sports. Through themes of teamwork and inclusion, the film challenges social stereotypes about Deaf individuals. The teachers and coaching staff at the Deaf school are all hearing individuals who bring the Deaf players to success.

In *Like for Likes*, a Deaf songwriter, Soo-ho, falls in love with a hearing woman, Na-yeon, as they work to overcome communication barriers. Through the love story of a Deaf-hearing couple, the film emphasizes mutual understanding and acceptance. While the two eventually find love, Soo-ho continues to be unable to communicate fully, highlighting the challenges of communicating with hearing individuals throughout the story.

*Midnight* centers on a Deaf woman, Kyung-mi, who becomes the target of a serial killer and must use her resourcefulness to survive. Although Kyung-mi demonstrates wit in navigating her ordeal, it is ultimately a hearing character, Jong-tak, who saves her, taking on the role of the hero.

*Silenced* is based on the true story of a hearing teacher who exposes abuse at a Deaf school and fights for justice for the students, criticizing society’s silence and corruption. A hearing teacher helps expose the abuse at a Deaf school, but no meaningful change occurs. In despair, one Deaf student attempts a murder-suicide with his abuser. Protests erupt afterward, yet the systemic issues remain unaddressed. Throughout the film, the focus is on the hearing teachers and the role they play in protecting or “saving” the Deaf students.

Finally, in *You’re So Precious to Me*, a struggling CEO named Jae-sik becomes the guardian of a Deafblind girl, Eun-hye, initially for financial reasons. Over time, he forms a genuine bond with her, changing his outlook on family and he decides to call himself her father. Jae-sik’s role as Eun-hye’s caretaker is the predominant focus of the film.

### Coding

In the analysis of movie clips with Deaf characters, a detailed coding system was applied to categorize both the involvement of Deaf individuals in the film production and the content of the films themselves. This comprehensive approach was divided into two primary levels: scene-level coding and movie-level coding. Scene coding was defined as coding for the portrayal of Deaf characters within the films by scene, while movie level was defined as coding the overall messages of the movie plots as well as the level of participation of Deaf people in development and production roles.

To code at the scene level, clips with Deaf characters from each sampled movie were entered into Nvivo, and the lines and dialogues of the characters were classified into two broad categories (cultural or medical) messages that didn’t clearly fall within cultural or medical categories were coded as general perspectives). Within each movie, the unit of analysis for coding was at the scene level. This was defined as a series of actions or lines performed in one location at one time point. Each scene was coded at both macro and micro levels to ensure comprehensive analysis. At the macro level, scenes were categorized into medical, cultural, and general perspectives, with multiple codes allowed per scene if they encompass various aspects. For example, a scene could reflect both medical and cultural perspectives, such as a Deaf character visiting a hospital and engaging in a cultural practice related to Deaf identity. At the micro level, each element within a scene—actions, dialogues, objects, places, emotions, and indirect expressions such as narrative tones—was counted based on its frequency of depiction. Narrative tones were analyzed through mood-setting elements like music, lighting, and camera angles to detect whether scenes conveyed messages of exclusion or inclusion. This involved an examination where every instance of these elements was tallied, such as repeated references to a hearing aid or the use of sign language. Dialogue and image descriptions were coded separately to enhance accuracy, ensuring that both verbal and nonverbal communications were thoroughly analyzed.

The categories, subcategories, and codes were based on codes used in Golos and Moses (2011) and Golos et al. (2021) but were modified for a media format (Table 1).

**Table 1:** Codes for cultural, medical, and general perspectives on Deaf characters in Korean movies

Category	Subcategory	Codes/Descriptors
Cultural perspective	Cultural interactions	Interaction with other Deaf; Referring to the Deaf community; Accepting Deaf characters as they are; Focusing on what Deaf can do; Using culturally related words
	Communication Strategies	Using visual strategies to get attention or interact; Using natural sign language
	Technology	Deaf technology (e.g., doorbell flasher, flashing lights, videophone, captioning)
Medical perspective	Disability	Focusing on what Deaf cannot do; Focusing on fixing deafness/wanting a Deaf character to be hearing; Deaf as lonely or isolated; Deaf character acting as if hearing; Pitying Deaf character; Labeling Deaf with a medical or disability term
	Communication Strategies	Deaf as perfect lipreader; Using spoken language only to communicate; Using manual communication that is not natural sign language
	Misconceptions about Deaf people	Deaf working and living in a negative or unhealthy environment; Deaf as angry; Deaf as poor; Deaf in danger; Deaf as sick; Deaf as noisy
	Technology	Talking about hearing aids; Getting cochlear implants
	Other Aspects	Lack of access to accommodation
General codes	General labeling	Mentioning the word “deaf” in regards to hearing level, as a general term or a Deaf character communicating without conveying any medical or cultural messages
	General Communication Strategies	Using the word “signing” or written language without using or mentioning KSL
	Services for the Deaf	Using or mentioning captioning, accommodations, hearing dogs, interpreters, etc.

*Note*. KSL = Korean Sign Language.

Characters who were DeafBlind were also included in the analysis. However, according to the current legal regulations in Korea (Kim, 2021, December 15), DeafBlind is recognized as a multiple disability of Deaf and blind. If a scene or character acknowledged a character was DeafBlind, it was included in the analysis. Scenes or characters that were depicted as blind but not Deaf were excluded from the analysis.

To code at the movie level for the plots where a Deaf character was a main character, we coded as: (1) medical messages about Deaf people were constant throughout the plotline including at the end of the film; (2) cultural messages about Deaf people were constant throughout the plotline including at the end of the film; or (3) there was transformation from the beginning to the end of the story from medical to cultural messages or cultural to medical overall perspectives of Deaf people.

For involvement in development and production at the movie level, movies were coded as to whether Deaf people were consulted in the film development or production including directors, writers, and actresses/actors of the movies. Several steps were taken to determine whether Deaf people were involved or consulted in the development or production of the film. These included searching through interview videos on YouTube with the production staff or actresses/actors to find out whether they are Deaf or not. The lead author also examined the movie’s commentary and ending credits to determine whether they acknowledged contributions or consultations by the Deaf community. Depending on whether or not Deaf people were reported as participating in the film’s production, the movie was classified into three categories: strong involvement, limited involvement, or no involvement. If one or more of the directors or the writers were Deaf, or the Deaf actresses/actors played roles of the Deaf characters, it was labeled as “strong involvement.” If there were no Deaf participants as directors, writers, or actresses/actors but had given consultations about it, it was classified as “limited involvement.” If no Deaf people were identified as being involved in the production, it was categorized as “no involvement.”

### Data analysis

To analyze the portrayal of Deaf characters and themes, the frequency of these codes was calculated both within scenes from each movie and across the six movies using Nvivo. Frequencies were calculated at multiple levels: across the three broad categories, the frequency of subcategories within each broader category (e.g., communication strategies, technology, misconception), and the frequency of individual codes (e.g., using natural sign language, talking about hearing aids, Deaf in danger) within each subcategory. Descriptive statistics were used to quantify these frequencies. This provided how often each category, subcategory, and individual code appeared. This involved counting the occurrences of each code in all scenes and calculating the mean and standard deviation to understand the data distribution.

### Positionality and interrater reliability

The lead author is a native speaker of Korean, fluent in KSL, and has firsthand experience working in the Korean Deaf community. The co-author, while involved in all aspects of the research study, is not Korean or fluent in KSL. As such, the lead author trained a second coder who is Korean Deaf and was raised in Korea. He is fluent in KSL and familiar with both Korean culture and Korean Deaf culture. The training involved practicing together on five movie clips, which were not used in the final analysis, to ensure a mutual understanding of the coding process and criteria. During these practice sessions, the lead author and the second coder discussed and agreed on the coding for each clip. After reaching a consensus, the second coder independently coded 50% of the data that were randomly selected to check interrater reliability. The results showed a 98.8% agreement in categories, 96.5% in subcategories, and 93.8% in individual codes. Any disagreements were discussed and resolved.

## Results

**Figure 1:**
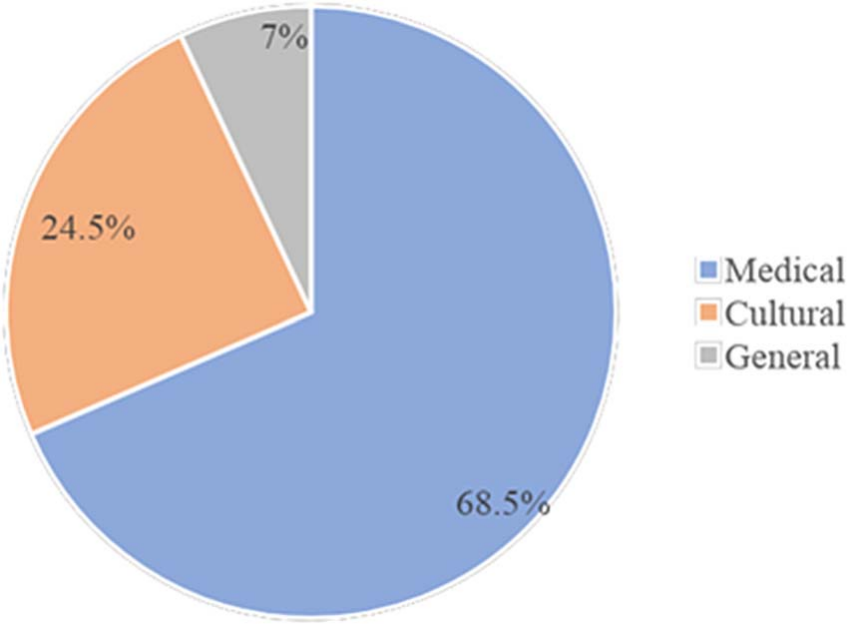
Distribution of portrayals of Deaf characters by the three broad categories.

Regarding movie results, there were a total of 1,609 references across the two broad categories (cultural and medical). Among them, the medical perspective was portrayed the most frequently across the six movies. There were 1,102 references to this perspective, representing about 68.5% of the broad categories found. Less often, the movies included references to depicting Deaf characters from a cultural perspective (*N* = 394; 24.5% of total references). Lastly, 113 references (7%) to a general category were found. The distribution of the three broad categories is illustrated in Figure 1.

**Figure 2:**
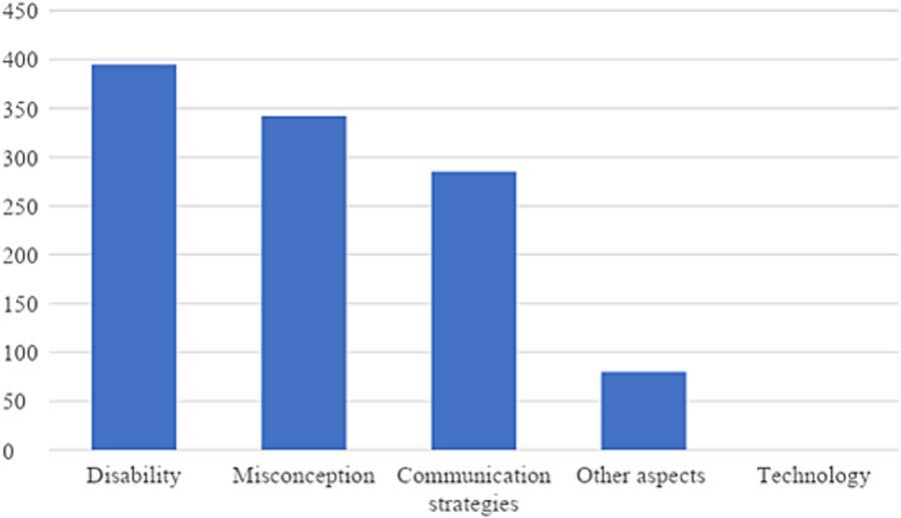
Total number of references to subcategories within the medical perspective category.

In the category of medical messages (see Figure 2), certain subcategories were referenced more than others. The subcategories “disability,” “misconception,” and “communication strategies” were frequently referenced, while the subcategories “other aspects” and “technology” were not. The subcategory “disability” was the most frequently referenced at 395 times, accounting for about 35.8% of the total medical perspective category. Also, the subcategory “misconception” was referenced 342 times (31%), and “communication strategies” were coded 285 times (25.9%). The subcategory “other aspects” were coded only 80 times (7.3%), and “technology” was not found.

Analyzing the codes within the subcategories related to the medical perspective, the code “Deaf in danger” (186 references, 16.9%) and “focusing on what Deaf cannot do” (176 references, 16.0%) were the most frequently found. They also portrayed Deaf characters as having conversations only through spoken language (155 references, 14.1%). Finally, the code “Deaf as lonely or isolated” was referenced 151 times (13.7%), describing the Deaf characters as socially isolated.

Codes that were rarely found include: “focusing on fixing deafness/wanting a Deaf character to be hearing” (0.6%) and “Deaf character acting as if hearing” (0.5%) were coded only seven and six times each. In addition, there was no mention of technology to improve hearing such as hearing aids and cochlear implants (Table 2).

**Table 2:** Total number and percentage of the most referenced codes within the medical perspective category

Subcategories	Code	Frequency	% within category
Misconception	d/Deaf in danger	186	16.9%
Disability	Focusing on what d/Deaf cannot do	176	16.0%
Communication strategies	Using spoken language only to communicate	155	14.1%
Disability	d/Deaf as lonely or isolated	151	13.7%
Communication strategies	d/Deaf as perfect lipreader	83	7.5%
Other aspects	Lack of access to accommodation	80	7.3%
Misconception	d/Deaf as poor	54	4.9%
Communication strategies	Using manual communication that is not natural sign language	47	4.3%
Misconception	d/Deaf working and living in a negative or unhealthy environment	40	3.6%
Disability	Labeling d/Deaf with a medical or disability term	37	3.4%
Misconception	d/Deaf as sick	25	2.3%
Misconception	d/Deaf as angry	22	2.0%
Disability	Pitying d/Deaf character	18	1.6%
Misconception	d/Deaf as noisy	15	1.4%
Disability	Focusing on fixing deafness/wanting a deaf character to be hearing	7	0.6%
Disability	d/Deaf character acting as if hearing	6	0.5%
Technology	Talking about hearing aids	0	0%
Technology	Getting cochlear implants	0	0%

Regarding the codes within the subcategories related to the cultural perspective (see Table 3), “using natural sign language” (108 references, 27.4%) was the most frequently found. Also, the code “referring about the Deaf community” was fairly frequently referenced (99 references, 25.1%). In contrast, the code “Deaf technology” (12 references, 3.0%) was less frequently coded. Also, “accepting Deaf characters as they are” (10 references, 2.5%) and “using culturally related words” (5 references, 1.3%) were less referenced than other codes.

**Table 3:** Total number and percentage of the most referenced codes within the cultural perspective category

Subcategories	Code	Frequency	% within category
Communication strategies	Using natural sign language	108	27.4%
Cultural interactions	Referring to the Deaf community	99	25.1%
Cultural interactions	Interaction with other d/Deaf	63	16.0%
Cultural interactions	Focusing on what d/Deaf can do	54	13.7%
Communication strategies	Using visual strategies to get attention or interact	43	10.9%
Technology	Deaf technology	12	3.0%
Cultural interactions	Accepting d/Deaf characters as they are	10	2.5%
Cultural interactions	Using culturally related words	5	1.3%

**Figure 3:**
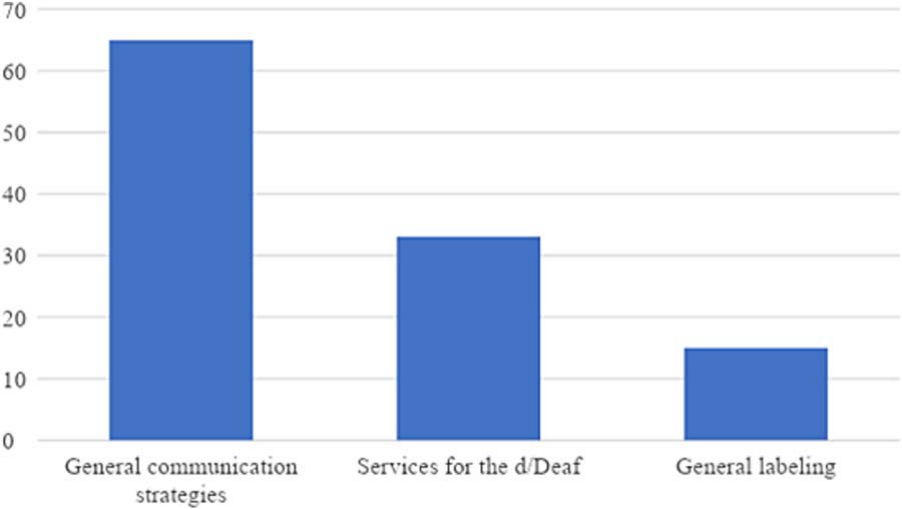
Total number of references to subcategories within the general perspective category.

Within the general perspective category (see Figure 3), the subcategory “general communication strategies” was referenced the most (65 references, 57.5%). “Services for the Deaf” was coded as the next most frequent code (33 times, 29.2%), while “general labeling” was found least often (15 times, 11.2%).

### Results by individual movies

When analyzing the frequency of cultural and medical categories for each movie (see Table 4), cultural and medical perspectives were referenced in all the movies with almost twice as many medical references. In each movie, the cultural perspective was referenced between 14 times and 178 times (i.e., *G-Love*), while the medical perspective was coded between 133 and 257 times for each movie with the highest number of references for the movie *Silenced*. Overall, all six movies except for *G-love* had between three to twelve times as many medical than cultural references.

**Table 4:** Total number of codes found in each movie within the cultural and medical perspective category

	Number of codes found	
Movie title	Cultural perspective	Medical perspective
Along with the Gods: The Two Worlds	21	136
G-Love	178	133
Like for Likes	15	133
Midnight	87	257
Silenced	79	272
You’re So Precious to Me	14	171
Total	394	1,102

### Overall messages in the portrayal of Deaf characters

In *Along with the Gods: Two Worlds*, the overall plot presents Deaf people from a medical perspective. There were 86.62% of medical messages and medical perspectives and this was constant throughout the movie. The Deaf character is portrayed as frail, impoverished, and reliant on others for care, and this continues throughout the movie. *Silenced* also presents a medical perspective with 77.49% of medical messages throughout the film. From beginning to end, it depicts Deaf students as victims of systemic abuse, with a focus on their helplessness and the necessity of external intervention, particularly through the hearing teacher’s actions. *Like for Likes* also presents a medical perspective. With 89.86% of medical messages, the Deaf songwriter depends on lipreading and text to communicate with the hearing woman throughout the film. The film emphasizes the challenges and limitations of Deafness as a barrier that complicates relationships and requires significant effort to bridge, even as the couple seeks mutual understanding. *You’re So Precious to Me* presents 92.43% of medical messages throughout the film. It continues to emphasize challenges and difficulties associated with raising and caring for a DeafBlind child.

In contrast, *G-Love*, however, presents a transformative plot. While there are 42.77% medical messages and 57.23% cultural messages, this emphasis from medical to cultural shifts throughout the film. Initially, the Deaf players are depicted as dependent on their hearing coach, reinforcing a narrative of reliance and framing deafness as a limitation requiring external intervention. As the plot progresses, the film shifts highlighting the Deaf players’ skills, teamwork, and resilience. It shows their accomplishments in sports, emphasizing the value of inclusion and the strength of the Deaf community, ultimately challenging societal stereotypes and celebrating Deaf culture.

### Deaf involvement in movies

In regards to Deaf involvement in the development and production of the movies, the lead author examined interviews with film directors, writers, actors, and commentaries and ending credits for each movie for information about the involvement of Deaf people in each movie (see Table 5). Based on the information found, it appears that all the selected six movies were made by hearing directors and written by hearing people. None of the six movies met the criteria for “strong involvement” of Deaf people in development and production and all of the actors who played significant Deaf roles were hearing. In the movie *Silenced*, Deaf actors were cast in minor roles, but the lead and supporting roles of Deaf characters were hearing.

**Table 5:** Movies that correspond to codes for each Deaf involvement

	Movie title(s)
Strong involvement	
Limited involvement	G-Love, Silenced
No involvement	Along with the Gods: The Two Worlds, Like for Likes, Midnight, You’re So Precious to Me

Two movies met the criteria of limited involvement. First, the movie *Silenced* acknowledged they received consultation from the Korea Deaf Research Institute, Onyang Sign Language Regional Children’s Center, Korea Deaf Association, the Deaf College Students Association, and the churches for the Deaf. Second, for the movie *G-Love*, members of the Korean Baseball Organization for the Deaf consulted on the storyline. It was also acknowledged that there was input from teachers and graduates of various schools for the Deaf.

However, the remaining four movies (*Along with the Gods: The Two Worlds, Like for Likes, Midnight,* and *You’re So Precious to Me*) did not show any involvement from Deaf people. In the movies *Along with the Gods: The Two Worlds* and *Midnight*, there were hearing interpreters on set who provided feedback on KSL to the actresses and actors. However, the lead author was not able to confirm the involvement of these staff other than the role of instructing KSL to the hearing actors. In the movies *Like for Likes* and *You’re So Precious to Me*, KSL was not used in the movies and there was no indication of consultations with Deaf people.

The frequencies of cultural and medical messages found in the films were compared according to the degree of Deaf involvement (see Table 6). Films with “limited involvement” had more cultural messages than those with “no involvement.” While films in both categories had many medical messages, those with no involvement had substantially fewer cultural messages than those with limited involvement.

**Table 6:** Average number of codes found in a movie regarding Deaf involvement and percentage of cultural perspectives in total codes

	Average number of codes found in a movie		
Deaf involvement	Cultural perspective	Medical perspective	% of cultural perspectives in total codes
Strong involvement	0	0	NA
Limited involvement	128.5	202.5	38.8%
No involvement	34.3	174.3	16.4%

Note: NA, Not Applicable.

The two films with limited involvement differ in their overall plot message. *G-Love*, which transitions from a medical to a cultural perspective, presents a more nuanced portrayal that incorporates elements of Deaf culture and emphasizes the strength of inclusion and the resilience of the Deaf community. In contrast, *Silenced* remains firmly rooted in a medical perspective throughout the plot. However, regardless of the level of Deaf involvement, stereotypes persist in the overall movie plots. Notably, films with limited involvement, such as *G-Love* and *Silenced*, focus on schools for the Deaf but still frame hearing characters as saviors, relegating Deaf characters to passive roles.

## Discussion

Based on the findings of this study, it appears Korean movies appear to convey more medical than cultural messages about Deaf people within the films and overall storylines. This is similar to previous studies analyzing Deaf characters in media and literature (Golos & Moses, 2011; Golos et al., 2021; Gangwish, 2017; Wolfe-Webb, 2021; Foss, 2014; Golos, 2010). The sampled movies typically portrayed Deaf characters as needing to be fixed, socially isolated due to their deafness or typically communicated through lip-reading. In five out of six analyzed movies (except for the movie *G-Love*), Deaf characters were overwhelmingly portrayed from a medical perspective, particularly as beings that need to become more like hearing people or be helped by hearing people. There is concern that this may negatively affect the self-perceptions of some Deaf adolescents who view the movies. Looking at the analysis of subcategories, the six selected Korean movies most frequently portray Deaf characters from the perspective of inability to function in everyday life and being described as being lonely, isolated, or unable to do things. In addition, they often contain misconceptions about Deaf people. For example, the movies portray Deaf people as being in danger, poor, or sick. If Deaf teenagers watch these portrayals, it may be difficult for them to accept and internalize the positive aspects of being Deaf or the lives they may be able to lead. This could potentially negatively impact the way they see themselves as a Deaf person.

The movies also repeatedly include communication strategies related to reading others’ lips, using spoken language, or simultaneous communication (i.e., Sim-Com; talking and signing at the same time). It is possible that these communication modalities could impact how Deaf adolescents view communicating through sign language after watching the movies. For example, they may mistake Sim-Com as a proper sign language. Moreover, they may believe that Deaf people should be able to understand spoken languages only through lip reading. These misconceptions can lead to the misunderstanding that sign language is subordinate to spoken language. These may have implications on the language choice and perception of languages for Deaf adolescents.

While movies may not directly state viewpoints, perspectives may be indirectly evident through the plot. For example, a movie might portray Deaf people from a medical perspective, but the director/producer’s perspective influences the messages that are conveyed. Looking at the overall plot helps to better understand the intentional messages in the storyline. It also provides insight into the director/producer’s awareness of Deaf people’s communication strategies such as Deaf people utilizing their various linguistic repertoires to communicate with individuals in different ways in different contexts.

For example, across three movies (*Silenced, G-Love,* and *Midnight)*, Deaf characters modify their linguistic repertoire depending on context and their interlocutors’ language abilities. Deaf characters display flexibility by adapting their communication styles in *Silenced*, Deaf characters use KSL with each other and supportive hearing characters, switch to Sim-Com with negative hearing characters, and use written language when needed. Positive-hearing characters respond by using KSL or Sim-Com, while negative characters use spoken language. In *Midnight*, Deaf characters use KSL with each other but expand to Sim-Com, writing, and spoken language when interacting with hearing characters. This demonstrates the potential awareness of the director or producer on the different ways Deaf people modify language in social contexts (De Meulder et al., 2019; Holcomb et al., 2023) and also has the potential to model this to Deaf adolescents viewing the movie as well.

In *G-Love*, the main Deaf character, who does not use KSL, instead uses spoken language, lip reading, Sim-Com, and writing with both Deaf and hearing characters. Deaf students other than the main Deaf character, however, use KSL among themselves and rely on interpreters or writing with hearing characters. As the main character never uses KSL and it is portrayed that his mother forced him not to use KSL, it may convey a message to views such as Deaf adolescents that spoken language/sim-com is a superior way of communicating than KSL.

Movies that consistently provide contradictory messages about Deaf people *within* the movies demonstrate a potential lack of awareness of the director/producer on the stereotypes depicted in the movie about Deaf people. For example, regarding the codes within the subcategories related to the cultural perspective, “focusing on what Deaf can do” was the most frequently found in the movie *Silenced* (e.g., a Deaf character can hear the sound of music). This result contrasts with the medical perspective category, where the code “focusing on what Deaf cannot do” was also frequently found in the same movie (e.g., it is emphasized that Deaf characters cannot hear).

These contradictory portrayals align with the overall plot, where Deaf characters are depicted as both capable and resilient yet ultimately reliant on hearing saviors for justice and safety. Despite moments that showcase their strengths, the film’s central focus on the hearing teacher as the hero reinforces the message that Deaf individuals cannot fully navigate their challenges without external assistance. This dual messaging risks perpetuating stereotypes, even as it attempts to highlight the capabilities of Deaf individuals.

Also, there were contradictory messages *across* movies and reflected in overall plotlines. While there were some instances of “referring to the Deaf community” across movies, there were more instances describing Deaf characters as socially isolated. These mixed messages being ongoing and in movies where there are no transformative aspects may create confusion in Deaf adolescents about perceptions of Deaf people and Deaf communities.

For instance, films like *Silenced* and *G-Love* touch on the existence of supportive Deaf communities within schools but simultaneously emphasize the isolation of Deaf characters, particularly in their interactions with the broader hearing world. While *G-Love* is transformational, shifting from a medical to a cultural perspective, it initially reinforces dependency by portraying hearing characters as saviors. Similarly, in *Midnight*, while the Deaf character displays resourcefulness, her struggle against societal isolation is a recurring theme. This dual portrayal of community and isolation risks reinforcing the idea that Deaf individuals, even within their own communities, are inherently marginalized and dependent on external support. Such conflicting narratives could influence how Deaf adolescents view their place within both the Deaf community and society at large.

Despite these issues, some films make strides toward highlighting resilience and inclusion. *G-Love* and *Like for Likes* challenge social stereotypes by depicting Deaf characters participating actively in sports and relationships. *Midnight* presents a Deaf character as a resourceful and intelligent character.

Lastly, none of the sampled films demonstrated a “strong involvement” of Deaf people in the making of the film. Even though they were movies about Deaf people, the director, writer, and actresses/actors were all hearing. While the films with “limited involvement” had substantially more cultural perspective codes than films with “no involvement.” They also had more medical perspectives than films with “no involvement” again providing mixed messages. Considering that even in the movies with “limited involvement,” the codes from cultural perspectives are less than half of the total codes it is possible that having “strong Deaf involvement” may impact the extent to which cultural messages are portrayed in films. This aligns with the recommendations of researchers (Golos & Moses, 2011), suggesting that having Deaf authors and illustrators may increase the positive cultural messages in books with Deaf characters.

This lack of strong Deaf involvement is evident in the overall plotlines. Films with no involvement such as *Midnight, You’re So Precious to Me, Along with the Gods: Two Worlds,* and *Like for Likes* had predominantly hearing perspectives, reinforcing narratives of Deaf dependence on hearing individuals. In contrast, films with limited involvement, like *Silenced* and *G-Love* still rely on hearing saviors, they emphasize the existence and value of the Deaf community. While lack of strong representation may have limited the potential for authentic cultural narrative, these findings suggest that greater involvement of Deaf individuals in the creative process may help shift the focus away from hearing saviors and foster more empowering, nuanced portrayals of Deaf characters.

Here too, it appears that cultural messages in Korean movies increased when the Deaf community was involved in film production. This suggests that movies with Deaf involvement in the development and production are more likely to portray cultural messages and developers and producers should be encouraged to collaborate with Deaf people throughout the film development and production process. It is possible that with increased Deaf involvement in the production, development, and acting, there may be increased portrayals of cultural perspectives with limited mixed messages.

## Limitations and future directions

Some limitations must be addressed in light of these findings. The main limitation is the limited sample size. Findings from the six movies may not reflect a broader scope of Korean movies that portray Deaf characters. More Korean films should be analyzed to have a better understanding of the portrayal of Deaf characters in Korean films.

Because none of the six selected films corresponded to “strong involvement,” it is challenging to determine the extent to which films with strong Deaf involvement have increased cultural messages. Therefore, further research on films and other media including Deaf people as active participants in film development and production is needed to better understand the impact of Deaf involvement.

Also, while the study gives attention to social stereotypes, biases, cultural diversity, and inclusiveness, it would benefit from further exploration of other factors such as economic influences, production considerations, audience needs and aesthetics, industry practices, and policy promotion. As movies are three-dimensional rather than two-dimensional, it is important to analyze the production and filming aspects as well such as camera angle and lighting.

Furthermore, as an East Asian country, Korea’s cultural context might differ significantly from that of Western countries. It is important to explore how these cultural differences affect the portrayal of Deaf characters in movies and influence audience perceptions. Analyzing these aspects could provide a more comprehensive understanding of the portrayal of Deaf characters in Korean films and suggest potential countermeasures for better representation.

Considering the potential to use media, including educational media as a supplemental resource to support identity development in home and school settings, it is important to examine all types of media for the portrayal of people. This includes print media as well as other types of video media including educational media.

Finally, it would also be important to study media from countries other than Korea as adolescents may have access to media more broadly. As the film industry becomes globalized, Deaf adolescents are exposed to various media worldwide. Movies and other media from all over the world may also impact the identity development of Deaf adolescents and all forms of global media should also be examined. So, conducting additional studies including a systematic review of the literature would be beneficial in expanding our understanding of what is known about the impact of Deaf people in films and other video and print media on the identities of Deaf adolescents at a global level. This foundational knowledge could also inform the recommendations for types of media educators and caregivers share with and recommend for Deaf adolescents in both home and school settings.

## Implications

The findings of this study provide potential implications for future Korean media development. First, more movies that portray Deaf characters from a cultural perspective are needed. Overall, it appears that Korean movies depict Deaf characters from a medical perspective rather than a cultural perspective which may contribute to negative perceptions of how Deaf adolescents see themselves as Deaf people (Byatt et al., 2023). If more movies portray Deaf people from a cultural perspective, it has the potential to positively affect the viewer’s sense of self-including self-esteem, identity development, and perceptions of how their language is valued (Cawthon et al., 2016; De Clerck & Golos, 2020; García-Fernández, 2014).

Importantly, future Korean media should actively seek Deaf participation when making films (Moses et al., 2018). Findings from this study indicate that movies with more Deaf involvement have demonstrated more cultural messages than those with no Deaf involvement. We recommend that future media adhere to the principle of “Nothing about us without us” (Franits, 2005), to provide accurate linguistic models and cultural perspectives. This also includes aligning recommendations for creating and using educational media (Moses et al., 2018) by having Deaf people directly participate in the development, production, and acting of films and other media.

While anecdotal, according to *The Making of Eternals* (Visnic & Hogan, 2022), Lauren Ridloff, a Deaf actress, pushed to use sign language in the film and assisted other actors in learning to sign. She also provided the guidelines by which the filmmakers could run a Deaf-friendly set. Moreover, according to actors, the movie *A Quiet Place* and *A Quiet Place 2* depicted Deaf characters and sign language in a more authentic manner by dedicating time to learning accurate ASL from ASL coaches and consulting Deaf people about their real-life experiences (Mendoza, 2021; Wolfe-Webb, 2021). Further examination of movies and other media is needed to determine the extent to which Deaf people taking part in development and production may lead to more cultural perspectives than those with limited involvement or no involvement.

## Conclusion

How Deaf people are portrayed in the media has the potential to provide powerful role models to Deaf adolescents. So, Deaf adolescents need opportunities to see Deaf people in media depicted from a cultural perspective to foster a healthy sense of self (Cawthon et al., 2016). However, deafness and Deaf people have typically been depicted in literature from a medical perspective, and the same appears to be true for the portrayal of Deaf characters in Korean movies (Foss, 2014; Wolfe-Webb, 2021; Golos & Moses, 2011). This research examined six Korean movies and identified a persistent pattern that presents negative messages about what Deaf people can and cannot do. Although one of the six films contained more messages from a cultural perspective than from a medical perspective, it also contained many scenes that had messages from a medical perspective. This could deliver a mixed message to Deaf adolescents. These patterns could contribute to Deaf adolescents forming a negative sense of self and feeling a disconnect from Deaf people and Deaf communities. Further exploration is needed to better understand the impacts of movies and media with Deaf characters on Deaf adolescents’ identity development.

Additionally, it appears Korean films limitedly include Deaf people in their productions. Deaf involvement in media could lead the film to have more accurate and positive portrayals of Deaf people including cultural perspectives. With increased cultural perspectives, it may enhance the Deaf adolescents’ pride and self-esteem as a Deaf person and a sense of acceptance and belonging to Deaf communities. Having a better understanding of the extent to which Deaf people are involved in media development as well the impacts of their involvement is needed on a global scale.

## Footnotes

1 For the purpose of this paper, capital “D” Deaf refers to those who identify as members of Deaf communities including Hard of Hearing, DeafBlind, DeafDisabled, and late-deafened people regardless of their hearing levels; “d” deaf and hard of hearing are used as general terms referring to the population of people with varying hearing levels.

## References for movies

Hwang (Director). (2011). *Silenced* [Film]. CJ Entertainment.

Kang (Director). (2011). *G-Love* [Film]. CJ Entertainment.

Kim (Director). (2017). *Along with the Gods: The Two Worlds* [Film]. Lotte Entertainment.

Kwon (Director). (2021). *Midnight* [Film]. CJ Entertainment.

Lee, & Kwon (Director). (2021). *You’re So Precious to Me* [Film]. Fine Story.

Park (Director). (2016). *Like for Likes* [Film]. CJ Entertainment.
